# Changes in the microbiota following allogeneic hematopoietic stem cell transplantation: A potential bioguide for clinical outcome?

**DOI:** 10.1016/j.htct.2025.106074

**Published:** 2025-12-12

**Authors:** Ekin Ece Gurer-Kluge, Fatma Savran Oguz, Zerrin Aktas, Sevgi Kalayoglu Besisik, Ugur Sezerman, Oral Oncul, Zafer Gulbas

**Affiliations:** a Istanbul University, Institute of Health Sciences, Istanbul, Turkey; b Istanbul University, Istanbul Faculty of Medicine/ Department of Medical Biology, Istanbul, Turkey; c Istanbul University, Istanbul Faculty of Medicine/ Department of Medical Microbiology, Istanbul, Turkey; d Istanbul University, Istanbul Faculty of Medicine/Department of Internal Medicine, Division of Hematology and Therapeutic Apheresis Unit, Istanbul, Turkey; e Acibadem University, Faculty of Medicine/Department of Biostatistics and Medical Informatics, Istanbul, Turkey; f Istanbul University, Istanbul Faculty of Medicine/Department of Infectious Diseases and Clinical Microbiology, Istanbul, Turkey; g Anadolu Health Center in affiliation John Hopkins Medicine/Department of Hematological Oncology, Istanbul, Turkey

**Keywords:** Intestinal microbiota, Blood diseases, Hematopoietic stem cell transplantation

## Abstract

**Introduction:**

This study aims to support our hypothesis regarding compositional changes in the intestinal microbiota by characterizing these changes through pre- and post-transplantation analyses. Additionally, it seeks to determine whether monitoring the intestinal flora could provide predictive or therapeutic insights into graft versus host disease.

**Methods:**

This study included adult patients who underwent allogeneic hematopoietic stem cell transplantation. Microbiota assessments were performed through stool analyses. Stool samples were collected twice: once before transplantation and once after engraftment. Following nucleic acid isolation, the samples were processed using New Generation Sequencing. Microbiota-associated pathways were examined using the Kyoto Encyclopedia of Genes and Genomes (KEGG) database. Statistical analyses were performed using R statistical software. In addition to microbiota analysis, resistance genes common in Gram-negative bacteria in the region (such as *OXA-48-like, KPC-like, NDM-like*, and *CTX-M-like*) were identified via classical polymerase chain reaction in stool samples collected after transplantation. The pathways were analyzed using the KEGG database.

**Results:**

Fifteen transplant recipients participated in the study. The Proteobacteria phylum increased in patients who tested positive for the *CTXM-1 group* and *OXA-48-like* resistance genes. *Blautia caecimuris* and *Enterococcus* exhibited significant changes following transplantation, while *Tyzzerella spp.* and *Dialister spp.* showed significant alterations after the onset of graft-versus-host disease. A marked change in *Eubacterium spp.* was also noted in patients with disease relapse. Two key metabolic pathways—acridone alkaloid biosynthesis and the D-arginine and D-ornithine metabolism—were associated with clinical outcomes.

**Conclusions:**

This study demonstrates that allogeneic hematopoietic stem cell transplants lead to significant alterations in intestinal microbiota composition, including increased pathogenic bacteria associated with graft-versus-host disease exacerbation. These findings suggest that microbiota monitoring may be a promising strategy for the prevention and treatment of graft-versus-host disease. Moreover, modulation of specific microbial metabolic pathways may influence disease clinical outcomes. As the first study of its kind conducted within the Turkish population, this research contributes novel insights to the existing literature and highlights the potential of microbiota-based approaches in post-transplant patient management.

## Introduction

Stem cells are undifferentiated cells that can self-renew and differentiate into specialized cell lineages, possessing the unique capability of unlimited cell division. Hematopoietic stem cells (HSCs) are responsible for regenerating blood and immune cells; their therapeutic potential is widely utilized in the treatment of hematological malignancies through stem cell transplantation. Allogeneic hematopoietic stem cell transplantation (allo-HSCT) aims to re-establish hematopoiesis from a donor-derived source and reconstruct a healthy donor immune system capable of eliminating residual tumor cells [1]. However, donor-derived immune cells—primarily alloreactive T cells—may also react against non-tumor tissues. Organs such as the skin, intestines, and liver are particularly susceptible to this response, leading to tissue damage and constituting a significant cause of morbidity and mortality following allo-HSCT. If the reaction occurs within the first 80–100 days after transplantation, it is called acute graft-versus-host disease (aGvHD) [2,3]. In allo-HSCT patients, conditioning regimens are used to ablate hematopoiesis, immunosuppressive therapies are administered to prevent the development of aGvHD alongside prophylactic antibiotic treatments aimed at reducing or preventing the risk of infections in this immunocompromised setting. The incidence and severity of aGvHD are associated with various risk factors [4].

Chemotherapy, given as a conditioning regimen, destroys epithelial cells and their integrity, resulting in bacterial translocation. The damaged epithelium secretes uric acid, adenosine triphosphate (ATP), and various proinflammatory cytokines. Pathogen recognition receptors such as ‘Toll-like receptors’ (TLR), ‘NOD-like receptors’ (NLR), and P2XR are activated by the pathogen-associated molecular patterns (PAMP) and damage-associated molecular patterns (DAMP). Antigen-presenting cells are activated. All of these reactions contribute to the development of aGvHD [5].

There is a suspicion of compositional changes in the intestinal microbiota after transplantation and also that the change in intestinal microbiota may have a role in aGvHD. Thousands of microbial species essential to human life have colonized the human body [6] with over 1000 different types of bacteria having been identified in stool samples. A high degree of variation is observed between individuals at the species level. The gut microbiome plays essential roles in human physiology, including food digestion, maintenance of the intestinal barrier, prevention of pathogen colonization, regulation of the gut–brain axis, and immune system development. The intestinal microbiota is influenced by external factors such as diet, lifestyle, environment, medications, and stress, which can significantly reduce bacterial diversity [7, 8, 9]. Through the production of short-chain fatty acids (SCFAs), the microbiota contributes to ATP synthesis, the production of vitamins B and K, the modulation of immune cells such as macrophages, and the regulation of immune responses [10]. Metabolic pathways are closely linked to cancer and other diseases, particularly after stem cell transplantation. The biosynthesis of acridone alkaloids is notable for its wide-ranging bioactivities, including cytotoxic, antibacterial, antiviral, anti-tumor, and enzyme inhibitory effects [11]. T cell activity highly depends on metabolic function, which is crucial for anti-tumor responses, however, the tumor microenvironment can impair T cell metabolism and function [12]. Arginine is a precursor of various compounds, such as nitric oxide, polyamines, creatinine, and urea. Ornithine, an intermediate in the urea cycle, contributes to immune regulation and is not incorporated into natural proteins [13]. Some of these metabolites also contribute to initiating and regulating immune responses.

This study aimed to document the microbiota before and after transplantation in allo-HSCT patients diagnosed with leukemia and lymphoma. Additionally, it seeks to determine whether monitoring the intestinal flora could provide predictive or therapeutic insights into GvHD.

## Method

Fifteen patients and fifteen sibling donors in the transplant preparation process were included in this study. Stool samples from patients were taken before the initiation of the preparation regimen and after engraftment. Stool samples from donors were taken before the mobilization regimen and before transplantation. All stool samples were stored under appropriate conditions (−80 °C) until being processed. The microorganism DNA isolation process was performed using the Spin Column Nucleic Acid Isolation Kit (ZymoBIOMICS DNA Kits, USA) in groups of ten in accordance with the manufacturer's recommendations. All samples were subjected to microbial nucleic acid sequencing using Oxford Nanopore Technology [14]. In addition, the e*xtended*-*spectrum β*-*lactamases* (ESBLs; *CTXM-1* group) genes which are the most common of the *Enterobacteriaceae* family in Turkey and resistance genes that make up the enzyme carbapenemase (*OXA-48-like, NDM* and *KPC*) and the presence of the *vanA* gene responsible for vancomycin resistance in enterococci were investigated in the stool samples of patients after transplantation [15,16]. The associated pathways were analyzed using the Kyoto Encyclopedia of Genes and Genomes (KEGG) database.

The volunteers (patients and healthy individuals) included in the study were informed and consent forms were signed after ethics committee approval. Study approval was obtained from the Istanbul University, Istanbul Faculty of Medicine Clinical Research Ethics Committee (Date: 19.07.2019, No: 146,386) and supported by Istanbul University, Scientific Research Projects Coordination Unit.

### Nucleic acid isolation

In the first stage, nucleic acid was isolated from samples using a stool nucleic acid isolation kit (ZymoBIOMICS DNA Kits, USA). For DNA isolation, various combinations of previously defined methods were tested. These included physical (sonication or bead disruption), chemical (SDS or CTAB), and biochemical (proteinase K, lysozyme) cell lysis techniques with the most effective method being subsequently determined. Following this, silica columns were used to separate DNA from protein molecules in the lysed cells, and RNA contamination was eliminated through the application of RNase. At the final stage of isolation, the DNA attached to the silica columns was dissolved in water without the DNase/Pyrogen and the nucleic acid concentration was determined using a spectrophotometer. DNA samples were selected with a minimum concentration of 10 ng/µL (preferably 50–300 ng/µL) to meet the following purity criteria: an OD_260_/OD_280_ ratio of 1.8–2.0 and an OD_260_/OD_230_ ratio of 2.0–2.2.

### Microbiome sequencing

A two-primer polymerase chain reaction (PCR) with rapid adapter binding chemistry was simplified following the instructions of the manufacturer with small changes, and the modified 5-end 16S rRNA gene amplicons were produced for adapter attachment after PCR. The 16S rRNA gene-specific forward primer (27F) and reverse primer (1492R) were used for amplification of the V1-V9 region of the 16S rRNA gene. PCR amplification of 16S rRNA genes was performed using ZymoTaq™ Hot Start PreMix (USA) in a total volume of 25 µL containing inner primer pairs (50 nM each) and outer primer mixture with barcode (3 %) from PCR Barcoding. The amplified DNA was purified using the AMPure® XP (Beckman Coulter), and was measured using a NanoDrop® 1000 (Thermo Fischer Scientific, Waltham, MA, USA) and (Qubit, Thermo Fisher, Waltham, MA, USA). A total of 100 ng of DNA was incubated for 5 min at room temperature with a 1 µL Rapid Adapter. The prepared DNA was loaded into the R9.4 flow cell (FLO-MIN106; Oxford Nanopore Technologies) and was sequenced. The MINNOW software version 1.11.5 (Oxford Nanopore Technologies) was used for data collection.

### Bioinformatics and statistical analysis

Sequencing was performed on a MinION device using Oxford Nanopore Technologies (ONT) FLO-MIN106D and 16S raw readings were obtained as fast5 files. The initial bioinformatics analyses were performed using ONT guppy version 5.0.11. Consensus arrays were created using bbtools 38.91, magicblast 1.6.0, and samtools 1.13. The NCBI blastn (version 2.0.12) was applied in preparation of the operational taxonomic unit (OTU) tables in accordance with the NCBI general 16S bacterial taxonomy reference dated 10/8/2021. The generated OTU tables were used to calculate alpha diversity using R Statistical Computing Language (version 4.04) (readr, phyloseq, microbiome, vegan, descr and ggplot2 packages). Statistical analyses also used R Statistical Computer Language version 4.0.4 and Rstudio IDE 1.4 (tidyverse, readr, xlsx and ggplot2 packages). Shapiro-Wilks normality testing showed non-normal distribution and therefore the Bonferroni correction method was used after performing the Wilcoxon Rank Sum or Mann-Whitney U tests thereby identifying the bacteria which showed statistical differences. In addition, the pathways associated with the KEGG database were examined.

## Results

All patients received transplants from fully HLA-matched (10/10) sibling donors.

The mean age of patients was 46.66 ± 14.30 years (20–69 years), and 44.40 ± 11.56 years (24–61 years) for donors. The gender distribution was 9/6 (M/F) in patients and 11/4 (M/F) in donors.

The most common indication for transplantation was acute myeloid leukemia, followed by myelodysplastic syndrome, non-Hodgkin lymphoma, primary myelofibrosis, and Hodgkin lymphoma.

Three patients received a myeloablative conditioning regimen, three received reduced-intensity conditioning, and the remaining underwent non-myeloablative conditioning. All patients received antimicrobial prophylaxis. In all cases, peripheral blood stem cells were used as the stem cell source.

The 100-day post-transplant survival rate was 80 % with aGvHD developing in 20 % of the patients. Complete chimerism was documented in 13 % of the patients who achieved engraftment.

The transplant-related protocols are given in Table 1.

**Table 1:** Transplantation protocols.

aGvHD Prophylaxis	CSA/MTX
	TAC alone or in combination with MMF
	CSA (or TAC) alone or in combination with MMF
	PTCy
Conditioning Regimens	Busulfan and cyclophosphamide
	TBI and cyclophosphamide
	Fludarabine, melphalan and ATG
	Treosulfan and fludarabine
Antimicrobial Prophylaxis	
Antibacterial prophylaxis	Fluoroquinolones
Herpes simplex virus prophylaxis	Acyclovir
*Pneumocystis jiroveci*	Trimethoprim-sulfamethoxazole
Fungal infection	fluconazole (Diflucan) for yeast

aGvHD: acute graft versus host disease; CsA/MTX: cyclosporin-A and methotrexate (Methotrexate); TAC: tacrolimus; MMF: mycophenolate mofetil (CellCept); PTCy: posttransplant cyclophosphamide; TBI: Total body irradiation; ATG: rabbit anti-thymoglobulin (ATG). Three out of 15 patients developed aGvHD (skin; skin and liver; intestine).

**Figure. 1:**
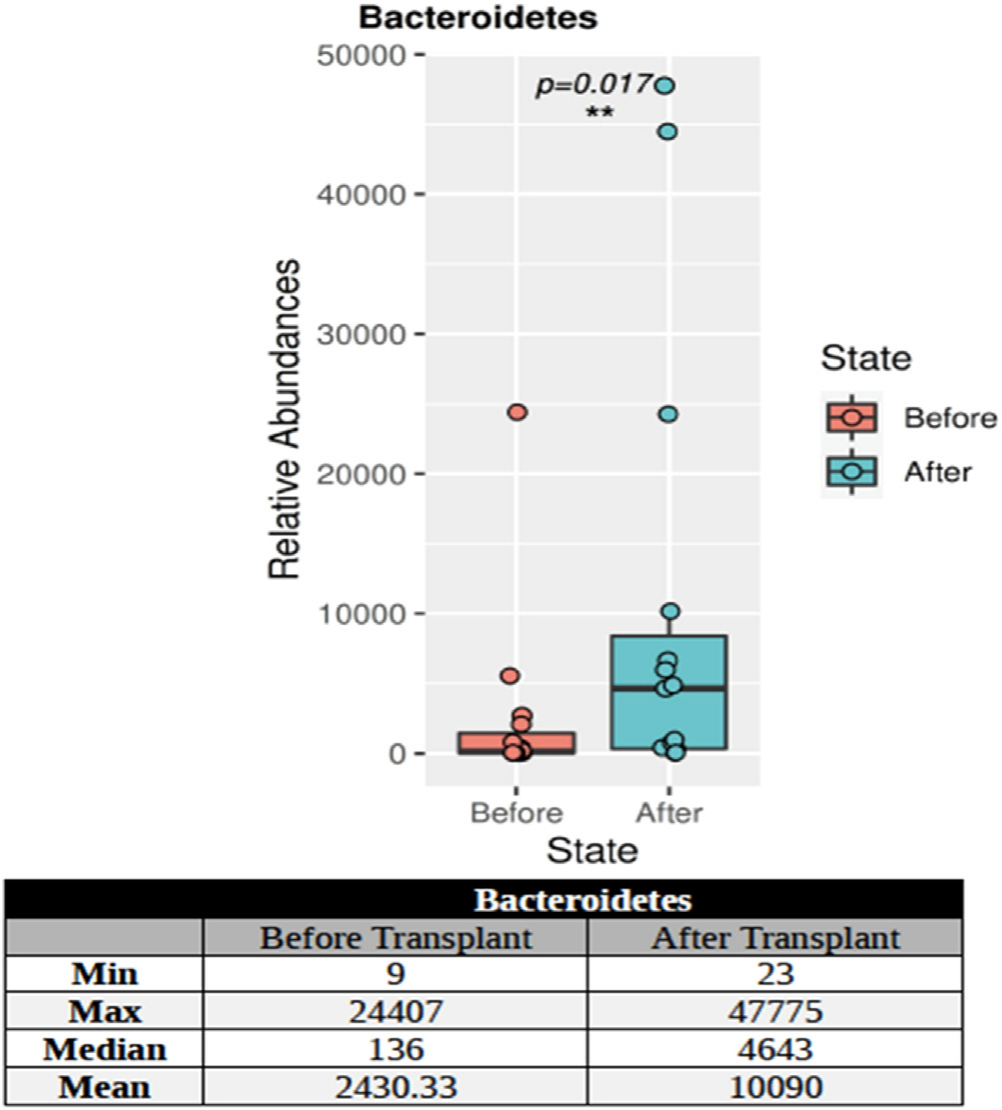
The change of the Bacteroidetes phylum before and after transplantation. The asterisk in the figure indicates that this phylum is a crucial feature, statistically different between the groups.

The *KPC* and *NDM* genes were not found in the post-transplant stool samples of the patients using PCR. *CTXM-1 group* and *vanA* genes were found in 13 % of the stool samples of patients and *CTXM-1* + *OXA-48-like* genes were detected in 13 %. 16S targeted sequencing phylum distributions of the intestinal microbiota of 15 patients with hematological malignancy before and after transplantation were calculated as percentage values. A total of 27 phyla were found. Only the Bacteroidetes phylum showed a statistically significant (p-value = 0.017) increase in individuals after transplantation (Figure 1).

**Figure. 2:**
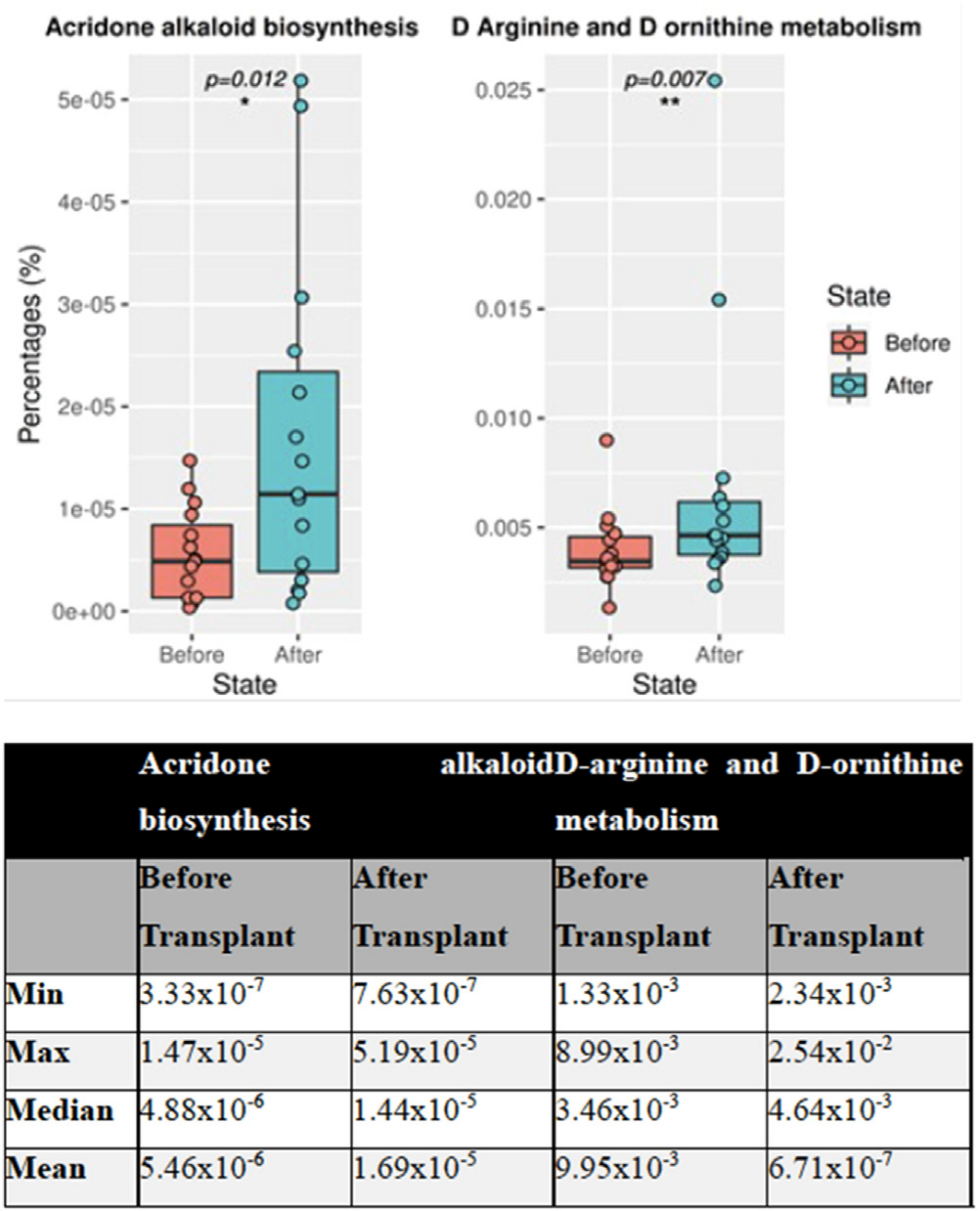
According to the KEGG database results, differences in Acridone alkaloid biosynthesis and d-arginine and d-ornithine metabolism. The asterisk in the figure indicates that this phylum is a crucial feature, which is statistically different between the groups.

A pathway analysis was performed after a 16S microbiome study of 30 stool samples from 15 patients before and after transplantation. Of 272 pathways, the obtained data showed statistically significant differences in two between the groups. These two pathways were found to be acridone alkaloid biosynthesis and d-arginine and d-ornithine metabolism (p-value = 0.012 and p-value = 0.007, respectively). A general increase was observed in acridone alkaloid biosynthesis (Figure 2) in the pathway analysis.

A total of 36 OTU differences were found in deceased and surviving patients within the first 100 days after transplantation. In the species-based analysis, significant decreases were detected in *Enterocloster bolteae* (p-value = 0.018), *Streptococcus salivarius* (p-value = 0.018), *Blautia caecimuris* (p-value = 0.018), and *Erysipelatoclostridium* spp. (p-value = 0.018). However, significant increases were detected in Faecalibacterium *spp*. (p-value = 0.02), *Enterococcus* spp. (p-value = 0.018), *Desemzia spp*. (p-value = 0.014), *Oceanobacillus spp*. (p-value = 0.007), *Brochothrix* spp. (p-value = 0.003) and *Anoxybacillus spp*. (p-value = 0.002).

**Figure. 3:**
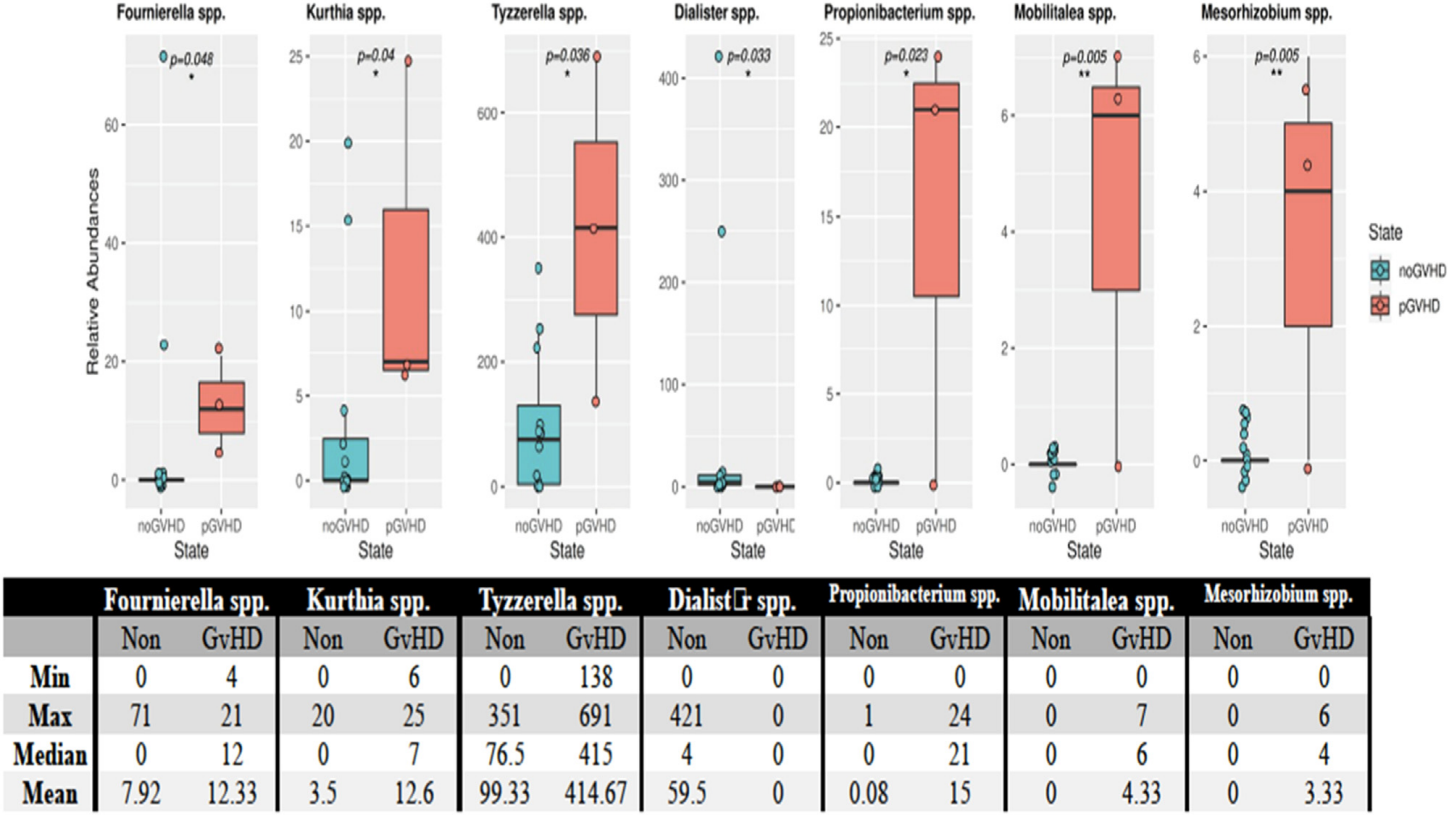
Species of bacteria in patients who developed and did not develop GvHD after transplantation. According to the Mann-Whitney U test the post-transplant minimum, maximum, median, and averages of the relative quantity values of the species *Fournierella* spp. (p-value = 0.048), *Kurthia* spp. (p-value = 0.04), *Tyzzerella* spp. (p-value = 0.036), *Dialister* spp. (p-value = 0.033), *Propionibacterium* spp. (p-value = 0.023), *Mobilitalea* spp. (p-value = 0.005) and *Mesorhizobium* spp. (p-value = 0.005) are given. The * and ** symbols in the figures show the statistically significant levels (p-value <0.05 and p-value <0.01, respectively).

Using the Mann-Whitney U test, seven OTUs showed differences in the microbiota between patients who developed and did not develop GvHD after transplantation. The bacterial species that were found to have statistically significant increases in patients who developed GvHD were *Fournierella spp*. (p-value = 0.048), *Kurthia spp*. (p-value = 0.04), *Tyzzerella spp*. (p-value = 0.036), *Dialister spp*. (p-value = 0.033), *Propionibacterium spp*. (p-value = 0.023), *Mobilitalea spp*. (p-value = 0.005) and *Mesorhizobium spp*. (p-value = 0.005) (Figure 3).

**Figure. 4:**
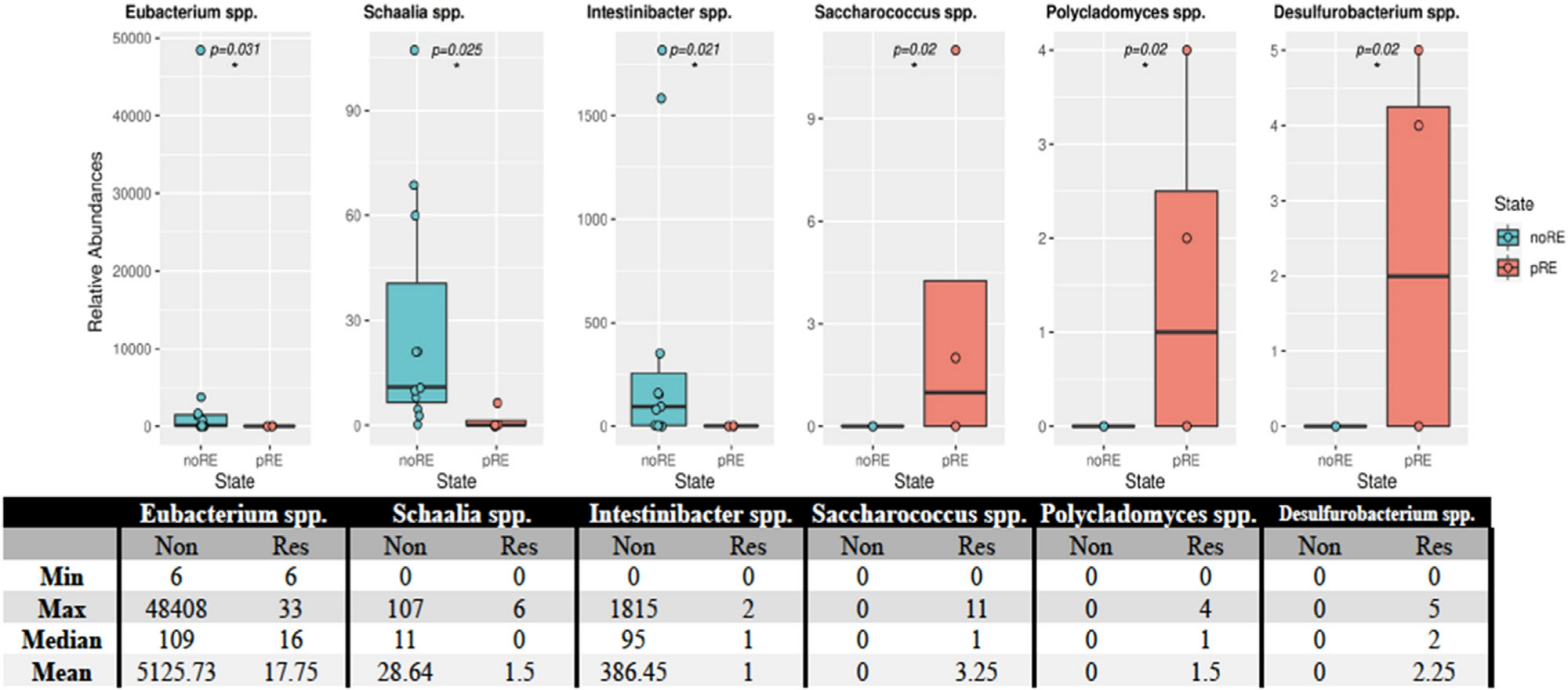
Distribution of bacteria in patients who developed relapse and those who did not. The relative abundances of the species that showed statistically significant differences are provided above. Statistically significant differences were observed for *Eubacterium* spp. (p-value = 0.031), *Schaalia* spp. (p-value = 0.025), *Intestinibacter* spp. (p-value = 0.021), *Saccharococcus* spp. (p-value = 0.02), *Polycladomyces* spp. (p-value = 0.02), and *Desulfurobacterium* spp. (p-value = 0.02). The asterisk (*) in the figure indicates a statistical difference between the groups.

Of the 26 phyla detected in 15 patients with hematological malignancies, the Actinobacteria phylum showed a statistically significant difference (p-value <0.018) in individuals who developed relapse after transplantation. This phylum was found in smaller numbers in this group. Analysis of the microbiota at the species level revealed statistically significant differences between patients who developed relapse and those who did not (Figure 4). These differences were observed for *Eubacterium* spp. (p-value = 0.031), *Schaalia* spp. (p-value = 0.025), *Intinibacter* spp. (p-value = 0.021), *Saccharococcus* spp. (p-value = 0.02), *Polycladomyces* spp. (p-value = 0.02), and *Desulfurobacterium* spp. (p-value = 0.02).

The bacterial distribution at the phylum level was studied by grouping patients according to the presence or absence of the resistance gene. Of the 26 phyla detected, the Proteobacteria phylum showed an increase in patients with the resistance gene, though this change was not statistically significant.

## Discussion

The greater understanding of the intestinal microbiota developed over the last 20 years has caused significant changes in how various diseases are followed up and treated. The role of microbiota in hematological malignancies has also been revealed [17]. The first hint that the intestinal microbiota affects GvHD dates back to the early 1970s [18,19]. It is impractical to follow the changes in all bacteria in the microbiota with the classically applied culture methods in today's technology as not all bacteria can be produced in vitro. Therefore, genomic studies are needed for the identification of bacteria. In microbiota studies, it is crucial to detect by molecular methods not only the bacteria found in normal flora but also bacteria resistance genes; other viral, fungal, and parasite species are also important. All microorganisms and their metabolites are in a state of balance with the host, however under some conditions, a disturbance leads to dysbiosis.

Due to budget constraints, this study conducted investigations only in terms of bacteria, and not of other microorganisms. Some bacteria in the intestinal microbiota acquire resistance genes against glycopeptide group antibiotics such as cephalosporins, carbapenems, or vancomycin. These bacteria can cause life-threatening, invasive infections in transplant recipients and other immunocompromised patient groups via endogenous spread from the gastrointestinal system.

During allo-HSCT, factors such as the conditioning regimen, nutrition, infections, and antibiotics disturb the balance of the intestinal flora [20,21]. Thus the diversity of microorganisms may decrease with changes that occur in the gastrointestinal tract of patients [22]. Poor intestinal diversity may affect engraftment, suggesting that gut microbiota may be an essential factor in the success or failure of allo-HSCT [23]. The loss of intestinal diversity is usually associated with the loss of *Clostridia* species known to produce SCFA by taking advantage of the fibers in food [24]. One of the most well-known SCFAs, butyrate, is recognized as the energy source of intestinal epithelial cells. In an experimental study using a mouse model, the abundance of *Lactobacillus* species in the intestinal flora decreased following hematopoietic stem cell transplantation (HSCT) and was associated with the development of GvHD. Researchers suggested that the microbiota can control the severity of intestinal inflammation by protecting against GvHD damage mediated by *Lactobacillus* species [25]. A recent article indicates that reduced amounts of butyrate are found in intestinal epithelial cells after allo-HSCT in mice, which may increase intestinal damage due to the development of GvHD [26]. In this study, considering the complications after the transplant, differences were identified in the Firmicutes phylum, which also includes *Lactobacillus*. In addition, the Bacteroidetes phylum showed a significant increase after transplantation compared to the levels before transplantation.

Microbiota analysis of stool samples obtained from patients after engraftment and before transplantation revealed a significant loss of diversity. Systemic inflammation of the gastrointestinal tract plays a vital role in the onset and exacerbation of GvHD. Therefore, the progression of GvHD with gastrointestinal involvement was more severe than with the other types of organ involvement [27].

The current research project showed an increase, albeit statistically insignificant, in *Enterococcus* and *Clostridium* species in one patient who died of intestinal GvHD. Also, qualitative changes in the microbiota are known as a result of allo-HSCT, especially the loss of microbiota diversity, which is characterized by the depletion of short-chain fatty acid-producing anaerobes [28]. Therefore, different protective and pathogenic components of the microbiota affect GvHD and survival following HSCT. Studies showed that loss of microbiota diversity was affected by both treatments and the development of GvHD [16]. In addition, an extensive prospective study targeting anaerobic bacteria in HSCT patients showed a regression in GvHD damage, indicating that some specific species in the microbiota may have beneficial effects [22,29]. Given these data, determining risk groups is essential for investigating the relationship between microbiota, stem cell transplantation, and GvHD development. Developing new follow-up and treatment algorithms is essential for graft survival.

In the present study, the incidence of aGvHD was 16 %. Dysbiosis associated with GvHD is characterized by the loss of overall diversity in the microbiota [30]. Essentially, a decrease was found in *Clostridia* species. Still, the change was not statistically significant in the stool samples of patients before and after transplant, and after engraftment. An increased presence, although statistically insignificant, of *Enterococcus* species bacteria was detected in patients who developed GvHD, including those in the current study. This study showed that the predominance of the *Enterococcus* population in the microbiota was significantly associated with severe aGvHD thereby indicating a causal role of *Enterococcus* in the pathogenesis of GvHD [31,32]. In a study conducted on a larger cohort of adults, the relative abundance of the Lachnospiraceae species and the decrease in *Blautia* species have been associated with decreased mortality rates from GvHD [33]. In the present case, a decrease was detected in Lachnospiraceae and *Blautia* species in the post-transplant samples of the recipients. However, no relationship was found with GvHD and mortality. In addition, the examination of microbiota differences between patients who developed GvHD and those who did not showed that a total of seven OTU showed differences between the individuals. In patients who developed GvHD, the OTU counts of bacterial some species (*Fournierella* spp., *Kurthia* spp., *Tyzzerella* spp., *Dialister* spp., *Propionibacterium* spp., *Mobilitalea* spp., and *Mesorhizobium* spp.) increased.

A total of 36 OTU differences were found in patients who died compared to those who survived within the first 100 days after transplantation. In the review conducted on the species, significant decreases were detected in *Enterocloster bolteae, Streptococcus salivarius, Blautia caecimuris, Erysipelatoclostridium* spp, while significant increases were detected in *Faecalicoccus* spp., *Enterococcus* spp., *Desemzia* spp., *Oceanobacillus* spp., *Brochothrix* spp. and *Anoxybacillus* spp. Of the 26 identified phyla found in patients who developed relapse within the first 100 days after transplantation, Actinobacteria was found to have a statistically significant lower number in relapsed individuals.

Conditioning chemotherapy administered before HSCT leads to prolonged neutropenia and damage to mucosal surfaces, facilitating the passage of microorganisms through these barriers and predisposing to bloodstream infection with the most common causes in HSCT recipients being enterococci and viridans group streptococci [34, 35, 36]. The results of one study showed that broad-spectrum beta-lactamase (GSBL) genes were positively correlated with *Klebsiella* species in samples taken from intensive care unit patients of eight hospitals in Turkey [37]. *KPC* and *NDM* gene positivity were not found in tests performed on the stool samples taken after transplantations however 13 % of *CTXM-1 group* and *vanA* gene positivity, and 13 % *CTXM-1 group* + *OXA-48-like* gene positivity were detected. In the current study, *Klebsiella* species were higher in number in patients who were detected to have resistance genes. In Turkey, *CTXM-1 group* and *OXA-48-like* resistance genes are widely observed, especially in *Klebsiella pneumoniae* strains [16]. Resistance to vancomycin develops due to the presence of *vanA* gene in bacteria of the *Enterococcus* species, which also leads to treatment failures. This study found the *vanA* gene in 13 % of patients. In patients, increases were detected in *Enterococcus* and *Klebsiella* species who died of septicaemia; however, these findings were not statistically significant.

*Citrobacter murliniae, K. pneumoniae*, and *Enterobacter cloacae*, which are known as hospital pathogens, are very important for the risk of infection in HSCT. Other members of the *Enterococcus, Citrobacter*, and Enterobacteriaceae families, such as *Enterobacter* and *Klebsiella*, are the most opportunistic members of the human intestinal microbiota. In nosocomial infections, *Citrobacter, Enterococcus, Klebsiella,* and *Enterobacter* are well-known possible sources and have been reported as the reason for the increase in morbidity and mortality rates [38]. Our results showed that colonization of the intestine with resistant strains was observed in some patients however, no nosocomial infections were detected.

Many metabolic activities have a crucial role in the state of health and disease. Various bioactivities of acridone alkaloids have been studied for the last 22 years [39]. Acridone, which is commonly found in healthy individuals, is also known to have cytotoxic and anticancer activity in addition to its antiparasitic and antimicrobial properties [40]. Other ongoing studies in mice with lymphoma suggest that acridone alkaloids are effective anti-cancer, and anti-proliferative agents [41]. Glutamate-consuming bacteria are predicted to utilize the acridone alkaloids pathway.

Another crucial metabolic pathway is the amino acid d-arginine, which is active in the body only in the ‘L’ isomer. l-arginine can be generated from the breakdown of proteins with ornithine being the central intermediate product in the arginine degradative pathway. Arginine and ornithine metabolisms are crucial in bacterial homeostasis [42]. d-Amino acids are found at high levels in humans and play a role in some biological functions. d-Amino acids may be present in some bacteria or may have adverse effects because they can be formed spontaneously in some reactions. According to a study conducted on mice, it was noted that the potential of the mitochondrial membrane is reduced after mitochondrial accumulation [43]. Again, in the same article, it was shown that d-ornithine caused no membrane potential changes. In addition, many studies have highlighted the potent anti-cancer activity of the acridone nucleus. Pathway analysis was performed after a 16S microbiome study of a total of 30 stool samples from 15 patients before and after transplantation in the present project. As a result of the obtained data, two of a total of 272 pathways showed statistically significant differences between the two groups. It was observed that these two pathways were acridone alkaloid biosynthesis and metabolism of d-Arginine and d-ornithine. The conducted pathway analysis showed that a general increase was observed in acridone alkaloid biosynthesis. l-arginine is a versatile amino acid that can be utilized as both carbon and nitrogen sources for bacteria and arginine can be de-novo synthesized by bacteria from several compounds, such as glutamate and glutamine [44]. l-arginine can also be metabolized by various enzymes such as Nitric Oxide Synthase or Arginase [45]. In this research, acridone alkaloid biosynthesis and d-arginine and d-ornithine metabolism pathways significantly increased after treatment. Considering all this information, this significant increase in both pathways may reflect the increase of a specific group of bacteria, such as glutamate-utilizing bacteria, and the increased expression of related pathways. This may suggest that these pathways have been functionally affected by the treatment and by some bacterial metabolites in the microbiota. It may be more relevant to evaluate all the data together.

The diversity of the microbiota community present in the environment before the HSCT procedure, the relative increases of saccharolytic commensals such as *Blautia* and *Fusobacterium nucleatum* includes risk factors for localized mucosal damage. Such microorganisms may have a greater chance of becoming a "pathobiome", such as a ‘pathogenic community adapted to becoming healthy’ during pre-HSCT hospitalization and the HSCT procedure, which cannot support immunological recovery in patients [46,47].

## Conclusion

In the future, supportive probiotics or prebiotics may be developed to increase the diversity of the commensal flora or control the gastrointestinal metabolome. Thus, these sensitive probiotics can be used as a biological key in patients with suspected bacteremia. Microbiota, which is regulated by non-pathogenic microorganisms, can even be used in patients who have undergone HSCT and have become immunocompromised. Dysbiosis after allo-HSCT can be treated with the enrichment of microorganisms required to prevent bacteremia and sepsis. In addition, a better understanding of the human ecosystem may allow the microbiota composition of patients to be used as a biomarker of disease. To exemplify, the microbial signature of patients may serve as a risk estimator for steroid-resistant GvHD, allowing them to start secondary treatments earlier. In general, microbiota-based therapeutics show great promise for preventing and treating GvHD and infections in patients after HSCT. It is essential to conduct further research aimed at developing targeted and individualized dysbiosis prevention and treatment regimens applicable to these patients.

This work was aimed to determine the changes in intestinal microbiota due to transplantation, and in treatment relapse, and the development of GvHD in patients with blood diseases.

The results of this study conducted in Turkey are correlated with those of similar studies in European countries and the United States. There is hope for therapeutic treatments with fecal transplantation, prebiotic support, and gut microbiota regulation for the treatment and prognosis of disease. Intestinal flora monitoring may provide guiding data on GvHD protection and/or treatment. Conducting the study with broader cohorts will contribute to the literature.

## Funding information

No funding source.

## Data availability

The data used to support this study's findings are included within the article.

## Author’s contributions

The authors indicated in parentheses made substantial contributions to the following research tasks: initial conception (E.E.G.K, F.S.O), design (E.E.G.K, F.S.O, S.K.B, Z.A, O.O), provision of resources (Z.G), collection of data (E.E.G.K), analysis and interpretation of data (U.S.), and writing and revision of papers (all researchers).

## Conflicts of interest

The authors declare no conflict of interest.
